# Pre-exposure to mechanical ventilation and endotoxin influence bacterial growth and immune response during experimental ventilator-associated pneumonia

**DOI:** 10.1186/cc14092

**Published:** 2015-03-16

**Authors:** J Sperber, A Nyberg, M Lipcsey, A Larsson, J Sjölin, M Castegren

**Affiliations:** 1 Centre for Clinical Research Sörmland, Uppsala University, Uppsala, Sweden; 2 Uppsala University, Uppsala, Sweden

## Introduction

Overproduction of nitric oxide (NO) is correlated with adverse outcomes in sepsis. NO is additionally a central part of the innate immune system defense against pathogens causing ventilator-associated pneumonia (VAP), which can complicate the clinical course during mechanical ventilation (MV). We hypothesized that pre-exposure to MV and systemic inflammation from endotoxin each would influence bacterial growth in lung tissue, based on an altered immune response in experimental pneumonia. We used a porcine *Pseudomonas aeruginosa *VAP model with ventilatory and inflammatory pre-exposures before inoculation to evaluate bacterial growth, development of lung damage, total NO production and inflammatory cytokine response.

## Methods

Three groups of mechanically ventilated pigs were subjected to experimental VAP for 6 hours with intrapulmonary 1 × 10^11 ^CFU *P. aeruginosa *at baseline. Two groups were pre-exposed to MV for 24 hours before bacterial inoculation: MV + Etx (*n *= 6, received endotoxin 0.063 μg × kg^-1 ^× hour^-1^) and MV (*n *= 6, received saline in equivalent volume). One group, Un (*n *= 8), started the experiment unexposed to both MV and endotoxin, directly from the initiation of VAP. Postmortem lung tissue samples rendered bacterial cultures. NO production was measured with urinary nitrate levels over 6 hours of VAP.

## Results

The animals pre-exposed to endotoxin (MV + Etx) displayed higher bacterial growth (CFU × g^-1^) (*P *< 0.05), lower PaO_2_/FiO_2 _(*P *< 0.05) and lower nitrate levels (*P *< 0.01) than the unexposed animals (Un). Plasma TNFα levels were higher in Un than in both pre-exposed groups MV + Etx and MV (*P *< 0.01). There were no significant differences between the two pre-exposed groups.

## Conclusion

Mechanical ventilation for 24 hours with concomitant endotoxin exposure enhances bacterial growth and lung damage during *P. aeruginosa *VAP, compared with inoculation without any pre-exposure to MV or endotoxin. The greater bacterial clearance in the unexposed animals was associated with higher NO production and higher levels of pro-inflammatory cytokines.

